# Science at risk? Considering the importance of academic freedom for STEM research production across 17 OECD countries

**DOI:** 10.1371/journal.pone.0298370

**Published:** 2024-02-15

**Authors:** Frank Fernandez, Volha Chykina, Yin Chun Lin

**Affiliations:** 1 University of Florida, Gainesville, Florida, United States of America; 2 Jepson School of Leadership Studies, University of Richmond, Richmond, Virginia, United States of America; 3 Graduate Institute of Technical and Vocational Education, National Taipei University of Technology, Taipei, Taiwan; Instituto Tecnologico Autonomo de Mexico, MEXICO

## Abstract

Since 2011, a declining trend in academic freedom globally has paralleled a rising tide of neo-nationalism. We use fixed effects models to examine data from the Varieties of Democracy (V-DEM) academic freedom index and bibliometric data for 17 OECD countries across nearly three decades (1981–2007) that precede the recent decline in academic freedom. We find substantial, statistically significant, positive relationships between cross-nationally comparable and longitudinal measures of academic freedom and volume of STEM publications. Additionally, academic freedom positively influenced the quality of STEM publications as measured by journal rankings. Our findings were relatively consistent across various measures of academic freedom and model specifications. We discuss implications for safeguarding academic freedom, applying neo-institutional theory, and identifying directions for future research.

## Introduction

While people around the world were distracted by the COVID-19 pandemic, India tipped from being classified as a democracy to being classified as an electoral autocracy [1]. With this development, the majority of the world’s population now lives in countries classified as electoral or closed autocracies. Only about 14% of people live in a country classified as a liberal democracy. The “third wave of autocratization” is punctuated by violations of international norms and human rights, including restrictions on free speech, media reporting, and other efforts to “intimidate and silence critics, and repress civil society organizations” [1].

Universities are becoming targets amid the new wave of autocracy and neo-nationalism [2]. In China, universities are crucial in Xi Jinping’s embrace of technological nationalism, yet Xi’s government led the charge in forcing a more ideological curriculum and cracking down on academics whose views do not align with that curriculum [3]. In Russia, Putin’s regime has slowly demolished the little university autonomy that used to be present, with the government’s neonationalist rhetoric often evoking Soviet values and nostalgia [4]. Even in a country such as the U.S., which is classified as a liberal democracy [1], efforts to limit academic freedom are building momentum across the states. For instance, legislation was introduced in Ohio that would necessitate that university professors who teach climate science include inaccurate or misleading counterpoints in their teaching [5]. Taken together, these examples are part of a trend in the decline of academic freedom around the globe. Cross-national measures show that between 2011 and 2021, academic freedom substantially declined in 19 countries that were home to approximately 37% of the world’s population. Only two countries (The Gambia and Uzbekistan) scored substantially higher on the academic freedom index in 2021 than in 2011 [6].

Even as many countries embrace neo-nationalism and assume an autocratic stance toward regulating higher education systems and restricting faculty members’ academic freedom, they seek to use universities to cultivate economic development through science, technology, engineering, and mathematics (STEM) fields [7]. While some may argue that “there may be nothing inherently political in the nature of hard science research,” others observe that neo-national and autocratic crackdowns have “had a perverse effect of politicizing the one area of academe that historically was a refuge from politics, the sciences” [3]. Although scholars have previously examined the global expansion of STEM research over the 20^th^ century [8], there is a lack of scholarship that empirically examines the relationship between academic freedom and STEM research production across countries and time.

The purpose of this paper is to examine whether cross-national, longitudinal measures of academic freedom influence national STEM research output. Specifically, we address the following research questions: *Is there a positive relationship between academic freedom and volume of research production*? and *Is there a positive relationship between academic freedom and quality of research production*? By analyzing available bibliometric data, we show that academic freedom is positively related to research production among 17 countries over 27 years. Drawing on neo-institutional theory [9–11], we show that, in addition to academic freedom, connectedness to world society (i.e., share of world trade) and size of national higher education systems (i.e., number of universities per capita) are influential for national STEM research production, even after controlling for measures of economic development and research and development (R&D) spending.

Our findings hold important implications for protecting academic freedom. As espoused in numerous international accords and conventions, academic freedom is inherently valuable and a necessary precondition for fulfilling universal, global human rights to education. Yet, from an instrumental perspective, we show that academic freedom is also important in terms of cultivating science production. Toward the end of the paper, we consider how our findings offer theoretical implications for neo-institutional theory. Additionally, we consider our contributions to the literatures on academic freedom, science production, and research policy.

## Literature review

Throughout the 20^th^ century, access to higher education expanded and facilitated the emergence of a global “schooled society” [12]. Higher education is not only affected by demographic changes and government policies, it influences social change [12]. The global expansion of higher education facilitated the creation, legitimation, and expansion of new forms of knowledge. Relative to classic subjects like the humanities, which declined in popularity and enrollments since the beginning of the 20^th^ century, the expansion of higher education led to a global boom in the social sciences [13, 14], as well as the creation of fields like women’s studies and business administration [12, 15].

Through all this change, STEM fields held their ground relative to other fields and did not experience the stark declines of the humanities or the explosive growth of the social sciences. Minor declines in natural science or some STEM subjects, such as math, were relative and not absolute [16]. Although STEM departments did not yield as many interdisciplinary offshoots as the social sciences (for instance, women’s studies), they did contribute to the formation of new interdisciplinary subjects, including multiple biomedical science subjects, such as cognitive science, neuroscience, and biotechnology [17]. Thus, when examining STEM research since the latter half of the 20^th^ century, it is important to consider a broad definition of STEM that includes health-related, biomedical fields.

In addition to the evolution of higher education academic disciplines, increased production of scientific knowledge has characterized the post-WWII era [9, 12]. Innovation sparked by scientific research has become a major contributor to many countries’ economies, leading to these countries becoming post-industrial societies where producing ideas is the main driver of the economy [7, 18–20], although there are some findings to the contrary [21]. Research production is not only a driver of economic development, but also a marker of the health and robustness of countries’ university systems. Universities regarded as strong research institutions attract talented students, faculty, and scientists. Because STEM research aligns with new technological advances, increasing university-based science production supports a positive feedback loop among university research capacity, scientific output, and economic growth [22].

Although some scholars predicted that universities’ influence would wane and private industry would begin to lead national STEM research production [23, 24], universities have produced or co-produced increasingly large shares of STEM research across countries [9]. Some scientists specifically choose to work in university-based labs because they have academic freedom and are not restricted by profit-seeking expectations. The professional autonomy that defines academic freedom allows university scientists to develop research agendas that are not profitable, whereas the private sector would prevent or prematurely end projects that are not perceived as potentially lucrative or cost-effective. In this way, university researchers become important partners for industry because they can incubate technological innovations without the same restrictions as industry-based research-and-development labs [25].

As global competition for producing larger shares of STEM research has increased, many governments have responded by adopting research excellence or incentive policies to spur a subset of their universities to accelerate STEM publication output. This group includes countries like China [26, 27] and Russia [28] that are simultaneously embracing neo-nationalism and autocratic control of higher education. While education researchers have argued that academic freedom has, at least in part, facilitated research production, the evidence behind these claims is mostly anecdotal [29, 30].

### Academic freedom and higher education

Scholars have long argued that academic freedom is essential to teaching and research, as it lies at the foundation of the university mission. Academic freedom is generally defined as the ability of a professor to have autonomy over teaching, research (including publishing), and intramural speech (e.g., shared governance discussions about how a university should be run) and extramural speech (e.g., addressing public policy or matters of public concern) without external control or fear of repercussion [31]. At an international convening in Dar Es Salam, Tanzania, scholars equated academic freedom with the “freedom to pursue truth and knowledge,” which is threatened by authoritarianism [32]. In Tanzania, scholars further stipulated that academic freedom requires employment security and protections against capricious dismissal by government actors. American scholars have identified multiple ways of conceptualizing academic freedom, includingas a legal concept, professional norm, and human right [33–35].

In 1997, the United Nations Educational, Scientific and Cultural Organization (UNESCO) adopted the *Recommendation Concerning the Status of Higher-Education Teaching Personnel*, which declares that the universal human right to education “can only be fully enjoyed in an atmosphere of academic freedom” [36]. The UNESCO recommendation calls on governments to recognize that academic freedom must apply to universities (free from external interference) and to individuals working within universities (freedom from government and university authority). Additionally, 171 United Nations (UN) member states became signatories to the International Covenant on Economic, Social and Cultural Rights (ICESCR), Article 15 of which requires that states “respect the freedom indispensable for scientific research” [37].

In 2011, the UN Human Rights Committee (HRC) issued guidance on interpreting the International Covenant on Civil and Political Rights. HRC explained that limits on freedom of expression “must be understood in the light of universality of human rights and the principle of non-discrimination” [38]. By 2020, the UN Special Rapporteur for Freedom of Opinion and Expression reminded the 75^th^ session of the UN General Assembly that “academic freedom enjoys fundamental protection not only in international human rights instruments but also at the regional level” [39]. Examples of regional authority on the importance of protecting academic freedom include the Charter of Fundamental Rights of the European Union, the European Court of Human Rights, the European Convention on Human Rights (Article 10), and the Council for the Development of Social Science Research in Africa’s (CODESRIA’s) Juba Declaration on Academic Freedom and University Autonomy. Writing amid the resurgence of neo-nationalism and international decline of academic freedom, the Special Rapporteur cautioned that when governments justify encroachments on academic freedom for the purpose of preserving culture, tradition, and morality, those claims “should be treated with scepticism and extreme caution” [39].

The Special Rapporteur’s report identified several recent instances where autocratic regimes have retaliated against scholars in ways that suppress academic freedom over research and publishing. For instance, in Turkey, academics have been dismissed when their scholarship does not align with the government’s agenda. The Special Rapporteur cited a country report to note that firing Turkish academics has resulted in a shrinking portfolio of research topics [39, 40]. Other academics were blacklisted, which barred them from “publishing research, attending conferences, and undertaking foreign travel” [39]. The political context in Turkey does not just prevent academics from disseminating research findings, widespread surveillance of teaching and research materials leads to self-censorship and prevents academics from pursuing research in the first place [39, 40].

In the schooled society, multiple international authorities have asserted that academic freedom is essential to the functioning of a modern university. This recognition reflects that universities have historically been safe havens for pro-democracy and pro-civil-rights movements. For instance, in the United States, the Civil Rights Movement gained ground on college campuses [41, 42]. Democracy movements also grew out of college campuses in mainland China and Hong Kong [43, 44], South Korea [45, 46], across Latin America [47, 48], as well as in support of South Africa’s anti-Apartheid movement [49].

However, this proclivity of universities to challenge the status quo and to criticize the existing power structures makes them a primary target of neo-nationalist leaders intent on suppressing dissent. Neo-nationalist leaders can rise to power in authoritarian states, such as China, and in democratic states, such as the United States and France (Douglass, 2021). These leaders build their political agenda around the idea of preserving dominant culture, even if it means stripping religious and ethnic minorities of their rights, and they relentlessly attack faculty and institutions when they do not support their agenda. When possible, neo-nationalist and autocratic leaders try to insert themselves into the education process, often demanding that universities change their curricula, adjust the topics that faculty can discuss, and provide funding only for research endeavors that support the regime.

For instance, the ideological revamping of university curriculum in China requires that faculty do not discuss such topics as civil society, press freedom, and universal values in their teaching [3]. Similarly, university students in Russia are discouraged from deliberating politically sensitive topics, and universities are being used to police student activism [4]. Restrictions on academic freedom are not limited to humanities and social sciences, though. In the United States, attacks on academic freedom have targeted science topics, such as evolution and climate change [50]. These infringements on academic freedom are not isolated cases but rather a part of a larger wave of pushback against academic freedom by neo-nationalist, illiberal leaders [2].

Many researchers have discussed the hypothesized link between research production and economic growth [7]. Some researchers [29] also have suggested that research production increases when scholars can research in the atmosphere of academic freedom, but to the best of our knowledge, none have tested the link empirically. Prior literature suggests several reasons research production might increase when scholars enjoy academic freedom. For instance, constraints on academic freedom limit which topics researchers can investigate [31], potentially inhibiting the originality of the work and the creativity of the authors. Additionally, when academic freedom is limited, researchers find it harder to secure funds to conduct their studies [2].

#### Academic freedom and research production

Many researchers have discussed the hypothesized link between research production and economic growth [7, 19, 20]. Some researchers have also argued that research production increases when scholars can research in the atmosphere of academic freedom [29, 30]. However, to the best of our knowledge, the link between academic freedom and research production has not been tested empirically.

Conversely, there are several reasons that research production might increase when scholars enjoy greater academic freedom. First, constraints on academic freedom limit which topics researchers can investigate [31], potentially inhibiting the originality of the work and the creativity of the authors. Additionally, limitations on academic freedom often entail regulating scholars’ collaborations with international colleagues, or unintentionally chilling scholars’ motivation to engage in such collaborations [2], which are essential to robust research activity [51]. In fact, at least one case study suggests that cross-country, co-authored research is more frequently cited than studies where all authors are from the same country [52]. Finally, when academic freedom is limited, researchers may find it harder to secure funds to conduct their studies [2].

## Theoretical framework

Neo-institutional theory suggests that mass research production is a global phenomenon [9]. Universities pursue legitimacy by adhering to the global model of research-active universities that seek to hire high-quality faculty, recruit excellent students, and produce research output at unprecedented rates [22]. Within and across countries, universities simultaneously compete and cooperate as they produce STEM research [9]. In this case, neo-institutional theory suggests that countries that are more connected to world society and follow long-standing international standards for academic freedom may be better able to contribute to global science.

We also draw on prior studies using neo-institutional theory to select variables to serve as proxies of countries’ connections to world society. For instance, following work by comparative sociologists [10, 11], we account for country-level proportion of world trade and a general measure of national democracy that may follow global democratization (or retrenchment to neo-nationalism). Within countries, we measure the strength of institutionalization of higher education by including university enrollment per capita and number of scientific organizations. Following a general approach in studies applying neo-institutional theory, we include economic indicators (GDP per capita and total government expenditures on research and development) in our models to estimate the independent influence of the connections to world society and institutionalization of higher education. Given the literature and theoretical framing above, we test the following hypotheses:

H1: There is a positive relationship between academic freedom and research production as measured by the number of STEM publications.
H2: There is a positive relationship between academic freedom and research production as measured by quality of STEM publications (i.e., journal ranking).

## Methods

### Data sources and variables

We addressed our research questions and tested our hypotheses by analyzing a comprehensive, cross-national panel dataset of national STEM publication output compiled from Clarivate’s Web of Science: Science Citation Index Expanded [51]. Specifically, we analyzed two outcomes: *STEM Publication Volume* and *STEM Publication Quality*. *STEM Publication Volume* was a continuous count of scholarly publications, while *STEM Publication Quality* was a recoded version of *STEM Publication Volume*, disaggregated by journal rankings. *STEM Publication Quality* measured counts of STEM publications in each of four quartiles of ranked journals and an additional category of unranked journals. We transformed the country-level dependent variables using the natural logarithm. Following prior studies on research output or research policy, we lagged the dependent variables by two years to acknowledge the delay between conceptualizing and publishing studies [53, 54].

We drew our key independent variable from V-Dem Institute’s Academic Freedom Index [55]. In developing the index, researchers focused on measuring academic freedom as a “multi-faceted” concept with foundations in international law [56]. In alignment with prior literature defining academic freedom and international norms (see above), V-Dem sought to compute measures that would be “(a) comparable across different university systems around the world and (b) specific to the academic sector” [56] including freedom to teach and research, freedom to speak and publish as scholars, and the freedom to critique government policies.

V-Dem’s historic *Academic Freedom* variable (v2clacfree) measures “freedom of academic and cultural expression” [57] using a Bayesian item response theory measurement approach [58]. Since this is an exploratory empirical study of the influence of academic freedom, we also substituted *Academic Freedom* with other, new variables from V-Dem’s Academic Freedom Index or academic space survey to test whether different elements of academic freedom also influenced STEM research production (see discussion in the Robustness section below). The alternate measures of academic freedom were Freedom to Research and Teach (v2cafres), Academics as Critics [of government policies] (v2cacritic), and Freedom of Academic Exchange and Dissemination (v2cafexch). See Tables 1–3 for descriptive statistics of the variables at the beginning, middle, and end of the panel.

**Table 1:** Descriptive statistics, 1981.

Variable Name	Obs	Mean	SD	Min	Median	Max
Univ. Enrollment per Capita	17	204.12	93.13	99.00	196.00	526.00
Number of Sci. Orgs	17	2.59	4.20	0.00	1.00	15.00
GDP per Capita	17	10973.46	3565.12	5371.17	11104.98	17679.87
Proportion of World Trade	17	2833.00	3083.81	330.00	1453.00	12825.00
Polity Index	17	9.76	0.56	8.00	10.00	10.00
Govt. R&D Spending–logged	17	6.80	1.59	4.46	6.60	10.43
Population–logged	17	2.71	1.17	1.24	2.29	5.44
Academic Freedom (v2clacfree)	17	2.50	0.60	1.44	2.44	3.53
Freedom to Research and Teach (v2cafres)	17	2.23	0.50	1.08	2.36	2.98
Academics as Critics (v2cacritic)	17	1.93	0.58	1.04	1.94	3.25
Freedom of Academic Exchange and Dissemination (v2cafexch)	17	2.22	0.60	1.05	2.45	2.97
Logged Number of All STEM Publications	17	8.64	1.29	6.56	8.65	11.78
Logged Number of STEM Publications in First Quartile Journals	17	7.78	1.41	5.63	7.71	11.28
Logged Number of STEM Publications in Second Quartile Journals	17	6.70	1.25	4.56	6.42	9.65
Logged Number of STEM Publications in Third Quartile Journals	17	6.02	1.15	4.23	5.93	8.75
Logged Number of STEM Publications in Fourth Quartile Journals	17	4.54	1.67	1.95	4.47	8.03
Logged Number of STEM Publications in Unranked Journals	17	7.43	1.27	5.06	7.42	10.19

**Table 2:** Descriptive statistics, 1993.

Variable Name	Obs	Mean	SD	Min	Median	Max
Univ. Enrollment per Capita	17	331.53	109.29	202.00	300.00	664.00
Number of Sci. Orgs	17	2.76	3.72	0.00	1.00	15.00
GDP per Capita	17	21672.72	6722.06	10401.98	22283.94	39270.11
Proportion of World Trade	17	3105.35	3516.45	297.00	1994.00	15051.00
Polity Index	17	10.00	0.00	10.00	10.00	10.00
Govt. R&D Spending–logged	17	7.65	1.46	5.20	7.63	11.15
Population–logged	17	2.78	1.18	1.27	2.35	5.56
Academic Freedom (v2clacfree)	17	2.86	0.52	1.44	3.08	3.53
Freedom to Research and Teach (v2cafres)	17	2.37	0.39	1.72	2.38	2.98
Academics as Critics (v2cacritic)	17	2.02	0.63	1.04	2.07	3.25
Freedom of Academic Exchange and Dissemination (v2cafexch)	17	2.37	0.46	1.55	2.46	2.97
Logged Number of All STEM Publications	17	9.17	1.19	7.14	9.19	12.05
Logged Number of STEM Publications in First Quartile Journals	17	8.67	1.23	6.52	8.73	11.67
Logged Number of STEM Publications in Second Quartile Journals	17	7.35	1.16	5.25	7.39	10.06
Logged Number of STEM Publications in Third Quartile Journals	17	6.26	1.07	4.98	6.00	8.86
Logged Number of STEM Publications in Fourth Quartile Journals	17	4.48	1.46	1.95	4.44	7.86
Logged Number of STEM Publications in Unranked Journals	17	7.34	1.16	5.43	7.35	9.94

**Table 3:** Descriptive statistics, 2007.

Variable Name	Obs	Mean	SD	Min	Median	Max
Univ. Enrollment per Capita	17	410.00	142.17	254.00	384.00	738.00
Number of Sci. Orgs	17	0.00	0.00	0.00	0.00	0.00
GDP per Capita	17	49457.09	13254.27	28863.97	47975.97	85139.96
Proportion of World Trade	17	2581.41	2736.57	367.00	1194.00	11851.00
Polity Index	17	9.88	0.49	8.00	10.00	10.00
Govt. R&D Spending–logged	17	8.39	1.24	6.82	7.93	11.68
Population–logged	17	2.86	1.19	1.48	2.40	5.71
Academic Freedom (v2clacfree)	17	2.93	0.37	2.25	3.08	3.53
Freedom to Research and Teach (v2cafres)	17	2.27	0.39	1.72	2.36	2.89
Academics as Critics (v2cacritic)	17	2.05	0.63	1.04	2.07	3.25
Freedom of Academic Exchange and Dissemination (v2cafexch)	17	2.34	0.46	1.55	2.45	2.97
Logged Number of All STEM Publications	17	9.84	1.02	8.36	9.67	12.35
Logged Number of STEM Publications in First Quartile Journals	17	9.53	1.03	8.07	9.41	12.08
Logged Number of STEM Publications in Second Quartile Journals	17	8.06	1.01	6.51	7.80	10.50
Logged Number of STEM Publications in Third Quartile Journals	17	6.90	1.06	5.52	6.54	9.25
Logged Number of STEM Publications in Fourth Quartile Journals	17	5.22	1.23	3.47	5.01	7.80
Logged Number of STEM Publications in Unranked Journals	17	6.16	1.03	4.74	6.06	8.69

We also included control variables based on our theoretical framework and prior empirical research [10, 11, 59, 60]. Specifically, we included country-level, time-varying measures of the strength of higher education as an institution. First, we controlled for university enrollment per capita because larger systems of higher education have more capacity to produce large volumes of research. Additionally, we controlled for the number of scientific organizations, which is an indicator of how organized a nation’s scientists and disciplines are if they form professional associations. Then, we controlled for each country’s share of global trade, which unlike a measure of economic output that measures country wealth or size, accounts for how connected a country is to the world society through the economic system. Connectedness to world society occurs through trade but reflects underlying political tensions (consider how a country like North Korea is relatively isolated from the international economic order based on political conflict). Just as connectedness to world society can be observed through trade, it can also be observed through the extent that a country’s government reflects international democratic norms and general policy liberalism; therefore, we controlled for level of democracy using the Polity IV scale.

To control for alternate, competing explanations of STEM research production, we included an annual measure for total population (logged), that might predict that countries with more people outproduce countries with fewer people. We also included a logged, inflation-adjusted measure of GDP per capita in U.S. Dollars [61], which would suggest that wealthier countries outproduce less wealthy countries (e.g., all else equal, more productive researchers may be drawn to live in wealthier countries). Finally, because research production could be influenced by central government spending on research and development, we controlled for the logged value of total government allocations for research and development measured as millions of U.S. dollars adjusted for inflation and purchasing power parity [62].

### Analytic strategy

After preparing the panel data, we estimated fixed effects regression models with year and country fixed effects and clustered standard errors. We used the Hausman test to determine that we would use fixed effects (as opposed to random effects) in our preferred models [63]. After preparing the panel data, we estimated fully specified models using a balanced panel of 17 countries with non-missing data between 1981 and 2007. The countries were: Australia, Austria, Belgium, Canada, Denmark, Finland, France, Greece, Ireland, Italy, Netherlands, Norway, Spain, Sweden, Switzerland, the United Kingdom, and the United States. Although academic freedom and research production variables were available for a broader set of countries, we could not obtain consistent data for GDP and R&D expenditures for non-OECD countries for the years we examined.

### Limitations

As with any study using observational data, our findings indicate long-term, cross-national correlations but do not imply causality. Additionally, our multivariate findings are limited to OECD countries with non-missing GDP and R&D expenditure data. Thus, we would use caution if generalizing our findings to non-OECD countries. Additionally, our analyses provide preliminary insights using Web of Science data, but the database is limited to English language publications and does not capture publications in non-English journals or outlets [51]. Further, the publication dataset does not capture more recent trends in STEM research production or the decline in academic freedom that is accompanying the more recent shift toward neo-nationalism. Future research may use more recent data, as well as non-STEM publications, to continue examining the relationship between academic freedom and research production. It is possible that our estimates about the influence of academic freedom on research production are conservative and that academic freedom may be even more influential for non-STEM publications. Finally, though we incorporate country-level fixed effects in our models, we cannot overcome the possibility of omitted variable bias, and future research may gather original data to control for the number of university faculty in each country across time.

## Results

Our findings support both hypotheses. After controlling for economic and higher education variables, a one-point increase in *Academic Freedom* correlated with a 7% increase in *STEM Publication Volume* (β = 0.07, *p* < 0.05). *Academic Freedom* also influenced quality of STEM publications. A one-point increase in *Academic Freedom* correlated with a 15% increase in a country’s STEM publications in academic journals ranked in the first quartile. Additionally, a one-point increase in *Academic Freedom* correlated with an 11% increase in a country’s STEM publications in academic journals ranked in the second quartile. The relationship was negative for publications in third-quartile journals and non-significant for publications in fourth-quartile journals. *Academic Freedom* was again statistically significant and positively associated with a 13% increase in publications in unranked journals.

In line with our theoretical framework, country-level institutionalization of higher education was generally positively and statistically significantly related to our research output measures. For instance, university enrollment per capita influenced overall STEM research production, as well as indicators of output in top ranked journals; conversely, it was not related to fourth quartile publications and was negatively related to publications in unranked journals. Similarly, number of scientific organizations was positively related to overall publications and highly ranked publications (but not related to publications in third or fourth quartile journals). Independent from academic freedom measures, connectedness to world society liberal democratic norms (polity index scores) had large, positive relationships with each outcome. See Table 4.

**Table 4:** Fixed effects models testing the relationship between academic freedom and research output for 17 countries, 1981–2007.

	Logged Number of All STEM Publications	Logged Number of STEM Publications in First Quartile Journals	Logged Number of STEM Publications in Second Quartile Journals	Logged Number of STEM Publications in Third Quartile Journals	Logged Number of STEM Publications in Fourth Quartile Journals	Logged Number of STEM Publications in Unranked Journals
Academic Freedom (v2clacfree)	0.07*	0.15***	0.11***	-0.13**	-0.19*	0.13*
	(2.44)	(4.31)	(3.38)	(-3.20)	(-2.43)	(2.39)
Univ. Enrollment per Capita	0.00025**	0.00056***	0.00022*	0.00063***	0.00022	-0.00073***
	(2.66)	(4.56)	(1.97)	(4.61)	(0.83)	(-3.81)
Number of Sci. Orgs	0.0073*	0.0124**	0.0090*	-0.0025	0.0050	0.0250***
	(2.03)	(2.66)	(2.06)	(-0.48)	(0.49)	(3.38)
GDP per Capita	0.0000024*	0.0000042**	0.0000032*	0.0000012	0.0000070*	-0.0000228***
	(2.11)	(2.87)	(2.38)	(0.76)	(2.21)	(-9.86)
Proportion of World Trade	-0.000011	-0.000045*	0.000009	-0.000043	0.000030	0.000252***
	(-0.71)	(-2.23)	(0.47)	(-1.95)	(0.69)	(7.97)
Polity Index	0.17***	0.18***	0.21***	0.24***	0.21**	0.24***
	(6.45)	(5.52)	(6.84)	(6.49)	(2.91)	(4.64)
Govt. R&D Spending (logged)	0.64***	0.88***	0.64***	0.31***	0.40***	-0.034
	(22.31)	(23.63)	(18.52)	(7.39)	(5.00)	(-0.57)
Population (logged)	0.20	0.46	0.09	0.84**	-1.59*	-1.49**
	(0.90)	(1.59)	(0.35)	(2.59)	(-2.53)	(-3.26)
Cons.	1.84**	-1.70*	-0.36	-0.44	4.10*	8.64***
	(3.10)	(-2.21)	(-0.50)	(-0.51)	(2.46)	(7.12)
*N*	459	459	459	459	459	459

*T* statistics in parentheses

* *p* < 0.05

** *p* < 0.01

*** *p* < 0.001

### Robustness

We re-estimated our analyses using alternate V-Dem measures of academic freedom and random effects models. Although the size of the relationships varied, our pattern of statistically significant results was relatively similar regardless of which variable we used from V-Dem’s academic freedom index. The pattern of results was similar to those presented above in models using the Freedom to Research and Teach (v2cafres) and Academics as Critics (v2cacritic) variables—with the latter having particularly large estimated effects on overall and first quartile publications. The Freedom of Academic Exchange and Dissemination (v2cafexch) variable had a generally negative relationship across outcomes, except unranked publications. See Table 5 for a summary of results from our robustness tests. Full results are available upon request.

**Table 5:** Summary of fixed effects models testing robustness of academic freedom measures.

	Logged Number of All STEM Publications	Logged Number of STEM Publications in First Quartile Journals	Logged Number of STEM Publications in Second Quartile Journals	Logged Number of STEM Publications in Third Quartile Journals	Logged Number of STEM Publications in Fourth Quartile Journals	Logged Number of STEM Publications in Unranked Journals
Freedom to Research and Teach (v2cafres)	0.08**	0.09*	0.17***	0.01	-0.21**	0.32***
	(2.87)	(2.49)	(5.18)	(0.11)	(-2.69)	(5.73)
Academics as Critics (v2cacritic)	0.25**	0.44***	0.22*	0.08	-0.11	-0.07
	(3.20)	(4.36)	(2.35)	(0.72)	(-0.52)	(-0.45)
Freedom of academic exchange and dissemination (v2cafexch)	-0.10*	-0.12*	0.01	-0.24***	-0.48***	0.43***
	(-2.31)	(-1.98)	(0.12)	(-3.81)	(-3.92)	(4.81)
*N*	459	459	459	459	459	459

*T* statistics in parentheses. Models include the same set of control variables as models in Table 1.

* *p* < 0.05

** *p* < 0.01

*** *p* < 0.001

## Discussion

Our findings hold important implications for safeguarding academic freedom. Academic freedom, as a right, merits protection without further conditions. Yet, we show that protecting academic freedom is important not only for humanistic reasons but also because countries that score higher on academic freedom indicators increase STEM research production over time. In addition to the correlation to a higher volume of STEM publications, academic freedom correlated to a higher quality of publications.

Spain serves as an important illustrative example of the broader trend in the data. In 1981 (the beginning of our panel), Spain was emerging from Franco-era autocracy and undergoing a “democratic transition” [64]. Although the country’s 1978 Constitution formally granted universities autonomy, more meaningful reform did not come until 1983 when legislation entrusted university rectors with autonomy and decentralized authority over teaching and research to academic departments [65]. V-Dem’s academic freedom data reflect these unfolding events and show an increase in academic freedom in Spain in the early 1980s. Spain’s higher level of academic freedom correlates with the overall increase in the nation’s research production into the 21^st^ century. To the extent that countries see STEM research production as an important factor in economic growth [7, 18–20], they should be alarmed by the rising trend in autocracy and declining trend in academic freedom across many countries.

Future research should examine countries that attempt to increase research production while repressing academic freedom. Our findings may have some transferrable implications for the STEM researchers around the globe with work cross-nationally and who have varying levels of access to academic freedom. Specifically, future research should examine the extent to which our findings have implications for China and other countries that are not included in our analyses. In other words, a case study may examine a country such as China, which recently surpassed the United States in research output [66]. Prior literature suggests that much of China’s progress came through co-authorship with the West, where academic freedom is relatively more protected. More than 40% of China’s scientific publications were co-authored with American individuals or institutions, and almost 95% of China’s scientific publications had co-authors in one of only 20 countries around the world—most of which were included in our analyses of OECD countries [67]. While China has increased research production while repressing academic freedom, it has accomplished its exponential growth in research production by fostering collaboration with liberal democracies. Future may examine the ways that China is still dependent on academic freedom elsewhere to achieve scientific research goals (i.e., through co-authorship with the West).

By showing that there is a positive relationship between academic freedom and STEM research production, this article makes an important contribution to the literature on academic freedom and human rights. It also contributes to the literatures on science production and research policy by providing empirical evidence that academic freedom should be considered as one factor that is worthy of equal consideration along with human capital or skill formation, economic wealth, and R&D expenditures. Additionally, it has implications for how researchers should think about the globalized nature of research production.

Neo-institutional scholars have long considered education a conduit for progressive cultural scripts, often embedded in school curricula that emphasize the primacy of human rights and civil liberties [12, 68–70]. However, recently these scholars have started to point to deviations from these scripts, especially in illiberal contexts. It appears that just as universities before used their power to spread progressive ideas, illiberal actors now adopt the same strategies to spread alternative scripts that fit their neo-nationalist agenda [71–73]. Further, it appears that these ideas diffuse across borders, thus creating a potential for institutionalizing a new, illiberal global culture. Because universities seek prestige and legitimacy and respond to national policy changes [74, 75], researchers should consider examining the ways that universities are responding to neo-nationalist and autocratic attacks on academic freedom that facilitates a research culture. For instance, drawing on ideas related to coercive isomorphism [76] and sociology of law [77], recent work indicates that university leaders adopt conservative policies and practices that go beyond what they are required to do by formal changes in the law so their universities can avoid governmental scrutiny and maintain prestige and legitimacy [78, 79].

Future research may use more recent data for a broader range of countries to continue to examine the relationship between academic freedom and national research output. Additionally, researchers may use non-STEM bibliometric data to examine the importance of academic freedom in other fields. The 20^th^ century gave rise to new subjects and witnessed massive expansion of the social sciences. It is conceivable that our results are conservative estimates of the importance of academic freedom And that academic freedom may be even more influential in the social sciences or humanities.

## Conclusion

As a concept, academic freedom is not just about individual rights. It exists as a public good to protect universities and researchers so they can pursue knowledge that benefits society. This paper offers evidence that at the macro level, threats to academic freedom matter. Repressing academic freedom may not just ‘chill’ individual faculty members’ speech, it may inhibit the global research enterprise in what is supposed to be the 21^st^-century knowledge economy.
